# Maltine

**Published:** 1880-03

**Authors:** 


### MALTINE.

PROF. YANDELL, in the *Louisville Medical News*, reports that after an extensive trial of the Maltine preparations of Reed & Carnrick, of New York, in private and dispensary practice, we are convinced that Maltine is one of the most valuable remedies ever introduced to the profession. Our exalted estimate of this article is confirmed by all of the many practitioners who have expressed to us their opinion of it. Wherever a constructive is indicated, Maltine will be found excellent. In pulmonary phthisis and other scrofulous diseases, in chronic syphilis, and in the various cachectic conditions it is invaluable. In convalescence it is a delightful and efficacious cordial. We have invariably found it liked by children, who devour it as they do candy. The Maltine Wine with Pepsin and Pancreatine has yielded us the happiest results in apepsia and atonic dyspepsia, and in general muscular and nervous debility. The preparations Maltine with Hypophosphites, Maltine Ferrated, Maltine with Pepsin and Pancreatine, and Plain Maltine we especially commend. It is prepared in innumerable combinations.

Maltine deserves to stand in the front rank of constructives; and the constructives, by their preventive, corrective and curative power, are probably the most widely-useful therapeutical agents that we possess.

